# Smoking and Quality of Life - Is there really an association? Evidence from a Nepalese sample

**DOI:** 10.1371/journal.pone.0221799

**Published:** 2019-09-06

**Authors:** Reshu Agrawal Sagtani, Sunaina Thapa, Alok Sagtani

**Affiliations:** 1 School of Public Health, Patan Academy of Health Sciences, lalitpur, Nepal; 2 Department of Dentistry, Annapurna Neurological Institute and allied Sciences, Kathmandu, Nepal; 3 Department of Oral and Maxillofacial Surgery, Kathmandu Medical College Teaching Hospital, Kathmandu, Nepal; Tribhuvan University Institute of Medicine, NEPAL

## Abstract

Tobacco smoking has a negative impact on human health. Thus, it seems plausible for it to affect perceived quality of life as well. Information in this regard is lacking in Nepalese context. Thus, a comparative study was designed to assess association of cigarette smoking with Health Related Quality of Life (HRQoL). This study was conducted among 125 current and never cigarette smokers who attended a teaching hospital in peri-urban area of Capital city of Nepal from December 2015 to June 2016. The data regarding their socio-demographic characteristic, tobacco history and HRQoL was collected using a self administered questionnaire. QoL assessment was made with the help of valid, translated version of WHO QOL-BREF. Results showed current smokers on an average used 4 sticks of cigarettes per day. Significant proportions of current smokers also consumed alcohol compared to never smokers (p <0.05). Mean difference of both overall QoL score and domain scores among both study groups were relatively small and thus, failed to reach statistical significance. On the other hand, the socio-demographic characteristics like male gender, currently earning and attaining more than higher secondary education were predictors of better HRQoL scores. According to study results, relationship between smoking status and self reported QoL is unclear. Thus, the policy makers should also focus on wider determinants of ill health and well being and not just smoking status. Further research is still needed to understand the effect of tobacco on self perceived health related quality of life.

## Introduction

Tobacco is an established risk factor for non-communicable diseases that have highest impact on disadvantaged and socially marginalized populations.[1–2].We find numerous studies which highlight the ill effects of tobacco on human health. These include cancer, lung disease, respiratory problems, coronary heart disease and stroke.[3] According to World Health Organization (WHO), tobacco consumption is one of the top ten risks in terms of burden of disease they cause. [4] Thus, it should be incorporated as an outcome measure in clinical care and research studies.[5]

In spite, of strong political commitment, more than 25000 deaths in Nepal is attributable to tobacco use. The prevalence of smoking among adult males is 37.1% while among females is 11.1%.[6] The National Demographic Health Surveys (DHS) between 2006 and 2016 show that smokeless tobacco use is common among males whereas among females smoking was more common than smokeless tobacco use. Both smoking and smokeless tobacco use were associated with older age and lower level of education.[7] In addition to mortality, tobacco has also been linked to morbidity and factors which affect quality of life such as nutrition and socioeconomic status.[8–9] Therefore, it seems plausible that smoking can be related to Health Related Quality of Life (HRQoL). HRQoL is a useful tool for examining the effects of smoking from the perspective of the individual as it covers a variety of domains that affect well—being of an individual. Evidence from developed countries suggest association of tobacco smoking with lower physical HRQoL and not mental HRQoL[10] while on the other hand independent statistically significant effect of smoking on all domains of HRQoL has also been observed.[11] (Smoking and economic). Persistent smokers with systemic health conditions like heart disease [12] and inflammatory bowel disease [13] also showed lower HRQoL compared to non-smokers with similar systemic conditions. In the South East Asia context, studies from Indonesia[14] and Taiwan[15] showed statistically significant association between smoking and HRQoL assessed through different tools. Alternatively, studies from both urban [16–17] and rural part [18] of Japan showed little or no association between them. As far as Nepal is concerned, we have no research exploring this association.

Nepal is a signatory to the treaty of WHO Framework Convention on Tobacco Control (WHO FCTC)[19] and has passed the Tobacco Product Control and Regulatory Act 2010 in November, 2011.[20] It has implemented the National Tobacco Control Strategic Plan in 2013–2016.[21] Recently, Tobacco Control Convention Strategy 2030 [22] was launched to provide guidelines for implementation of tobacco control programs in Nepal. Thus, Nepal is committed to tobacco prevention and control through directives like large health warnings in the packaging and enforcing smoke-free public spaces and transport Along with it, health professionals are encouraged to enquire about tobacco use, support for quitting habit and provide appropriate treatment and referral for patients in health care institutions.

This study aims to find association of cigarette smoking with HRQoL from a sample of Nepalese population. If we can provide evidence that cigarette smoking is associated with HRQoL along with being a major cause of disease and disability, it can be used as an additional rationale for intensifying tobacco control efforts in Nepal.

## Materials and methods

A cross sectional study was conducted among patients who came to Department of Oral and Maxillofacial Surgery of Nepal Medical College Teaching Hospital (NMCTH), Jorpati, Kathmandu for removal of tooth/teeth. This is a private teaching hospital and all the paying patients were only included in the study. The patients with severe pain and inability to respond were not included in the study. The study participants were divided into two groups i.e. *current smokers* who have smoked 100 cigarettes in his/ her lifetime and who currently smoke cigarettes. The other group comprised of *never smokers* who have never smoked, or who have smoked less than 100 cigarettes in his/her lifetime. The data was collected over the period of six months (December 2015—June 2016) with the help of self-administered questionnaire. The questionnaire comprised of information related to socio demographic variables, alcohol history, history of cigarette usefor the current smokers and HRQoL items.

World Health Organization Quality of Life Brief version (WHOQoL-BREF) was used for quality of life measurement. It is a self-reported questionnaire comprising of 26 items, each item indicating one aspect of life that is considered to have a contribution to a person’s quality of life. Twenty-four items measure four broad domains, namely physical health (7 items), psychological health (6 items), social relationships (9 items) and environment (8 items). Two other items measure the overall perception of quality of life and general health. Thus, 24 items constitute the four WHOQoL domains (physical, psychological, social, and environment). Since, WHOQoL-BREF employs a 5-points scale (1 to 5) the highest score (100) is achieved when no limitations or disabilities are observed. Higher scores indicate a higher level of self-perceived quality of life. The WHOQoL is available in Nepalese version and has been validated in Nepalese settings.[23]

A total of 250 patients i.e. 125 current smokers and 125 never smokers were invited to become a part of the study through non- probability sampling technique. The sample size was calculated taking reference^7^ from a study conducted in Indonesia using the following formula,

$$(n)=[2\sigma^{2}(Z_{\beta }+Z_{\frac{\alpha }{2}}{)}^{2}],$$

(effect size)^2^

where,

Level of significance (α) = 95%

Power of study (β) = 80%

Design effect of 2 was used and amplification by 10% for non-response error was done to reach the final sample size of 125 in each arm.

Descriptive statistics like mean, median, standard deviation range etc. were calculated for socio demographic characteristics, tobacco and alcohol history and HRQoL depending on the distribution of study variables. Domain wise HRQoL scores were compared between current and never smokers using Independent t test. Association between socio-demographic variables and alcohol use with HRQoL domains was tested with the help of Independent T test, Chi squared test, Analysis of Variance and post hoc comparisons wherever applicable. Predictors for HRQol were ascertained with the help on multivariate linear regression analysis after checking for multi-collinearity statistics. Informed written consent was taken from all the study participants. This study was granted ethical approval from the institutional review board (Research and Ethics Committee) of Nepal Medical College Teaching Hospital.

## Results

A cross—sectional study was done among 125 current and 125 never cigarette smokers who attended dental department of Nepal Medical College Teaching Hospital. A total of 129 current smokers and 126 non-smokers were approached and 125 in each category agreed to participate in the study. Table 1 shows that majority (32.4%) of the study participants belonged to the age group of 45–60 years with average age being 46.6 years. More than fifty per cent of the respondents were female (56.0%), currently married (88.8%) and not earning (61.6%). More than one thirds, (440%) of the respondents had not received any formal education. Stratified results showed that the current cigarette smokers had significantly higher mean age compared to non- smokers with p value = 0.003. Gender was also significantly associated with cigarette smoking status with higher proportion (61.6%) of males being current smokers. Current marital and earning status also found significant with smoking status with higher proportion of current smokers being married and earning. Furthermore, alcohol consumption was significantly associated with current smoking status with p value of 0.04. The average quantity of smoked cigarettes was 4 sticks per day. The average duration of cigarette smoking was 17 years. Among the smokers, 08.8% per cent of patients were also using smokeless tobacco. (Table 1)

**Table 1:** Description of the study population. (n = 250).

Characteristics	Categories	Current Smokers (n, %)	Non–smokers (n, %)	Total (n, %)	P value
**Age**	15–30 years	11 (8.8%)	30 (24.0%)	41 (16.4%)	<0.001*
	30–45 years	28 (22.4%)	43 (34.4%)	71 (28.4%)	
	45–60 years	56 (44.8%)	25 (20.0%)	81 (32.4%)	
	≥ 60 years	30 (24.0%)	27 (21.6%)	57 (22.8%)	
**Mean Age ± sd**		49.6 ± 13.8	43.46 ± 18.32	46.6 ± 16.5	0.003#
**Gender**	Male	77 (61.6%)	33 (26.4%)	110 (44.0%)	<0.001*
	Female	48 (38.4%)	92 (73.6%)	140 (56.0%)	
**Marital Status**	Currently married	116 (92.8%)	106 (84.8%)	222 (88.8%)	< 0.045*
	Currently unmarried	9 (7.2%)	19 (15.2%)	28 (11.2%)	
**Earning Status**	Currently earning	56 (44.8%)	40 (32.0%)	96 (38.4%)	<0.037*
	Currently non-earning	69 (55.2%)	85 (68.0%)	154 (61.6%)	
**Education**	No formal education	47 (37.6%)	63 (50.4%)	110 (44.0%)	0.115*
	Up to high school	29 (23.2%)	21 (16.8%)	50 (35.7%)	
	More than high school	49 (39.2%)	41 (32.8%)	90 (64.3%)	
**Alcohol Use**	Yes	29 (23.0%)	12 (9.6%)	41 (16.4%)	0.006*
**Tobacco History among current tobacco users (n = 125)**					
**Currently Smokeless tobacco user**			11 (8.8%)		
**Median duration of tobacco smoking**			16 (5–30) years		
**Median number of smoked cigarettes**			4 (2–5) sticks per day		

*Chi squared test used

# Independent T test used.

As shown in Table 2, the non- smokers have higher mean HR-QOL scores in all the 4 domains. However, the mean difference in scores between the groups was very small. Thus, it failed to reach statistical significance which indicates that current smoking status may not affect item response in HR QoL scale.

**Table 2:** Mean (sd) health related- quality of life (HR- QOL) by smoking status.

QOL measures	Never smokers (mean ± sd)	Current smokers (mean ± sd)	Mean Diff.	95% Conf. interval		P value#
				Lower	Upper	
**Rating of QOL**	3.18 ± 0.61	3.14 ± 0.56	0.040	- 0.106	0.186	0.07
**Satisfaction with health**	3.20 ± 0.62	3.30 ± 0.66	-0.096	- 0.256	0.064	0.08
**Physical domain**	60.43 ± 10.89	60.02 ± 11.80	0.416	-2.413	3.245	0.772
**Psychological domain**	58.49 ± 9.25	57.98 ± 8.70	0.504	-1.734	2.742	0.658
**Social domain**	62.49 ± 14.22	61.90 ± 13.30	0.588	-2.849	4.025	0.736
**Environmental domain**	56.95 ± 11.80	56.50 ± 9.60	0.456	- 2.225	3.137	0.738

#Independent T test used.

We tested association of demographic characteristics with four domains of HR-QOL. As shown in Table 3, we can see that males had significantly higher mean scores in the physical domain. In this study, currently earning patients reported significantly higher mean QOL scores in physical, psychological and social domain. It was observed that there was an increasing trend of mean QOL scores among the patients with higher educational qualification. The difference in scores reached statistical significance indicating that higher education is associated with improvement in mean QOL scores.

**Table 3:** Association of demographic characteristics with domains of HR-QOL.

Characteristics	Categories	Physical (mean ± sd)	Psychological (mean ± sd)	Social (mean ± sd)	Environmental (mean ± sd)
Age group (years)#	15–30	59.7± 10.9	58.7 ± 7.6	60.8 ± 14.9	55.1 ± 10.9
	30–45	61.7 ± 10.9	58.7 ± 8.4	63.9 ± 13.7	57.5 ± 11.3
	45–60	61.1 ± 11.7	59.2 ± 9.1	62.7 ± 12.6	57.5 ± 09.5
	≥ 60	57.5 ± 11.3	55.8 ± 9.8	60.7 ± 14.8	55.6 ± 11.5
Gender##	Male	*62.01 ± 12.2	59.37 ± 9.8	63.19 ± 13.8	57.37 ± 10.7
	Female	58.82 ± 10.4	57.34 ± 8.2	61.64 ± 13.9	56.21 ± 10.8
Current Marital status##	Married	60.45 ± 11.2	58.26 ± 09.1	62.86 ± 13.5	57.00 ± 10.6
	Unmarried	58.39 ± 12.1	58.04 ± 07.7	58.04 ± 15.9	54.50 ± 11.9
Current Earning status##	Earning	*64.14 ± 11.0	*60.78 ± 08.8	*64.92 ± 13.6	58.16 ± 09.8
	Not earning	57.79 ± 10.9	56.65 ± 08.7	60.71 ± 13.8	55.83 ± 11.2
Education level#	No formal education	57.78 ± 11.3	56.59 ± 9.2	60.95 ± 13.8	54.8 ± 11.6
	Up to high school	61.18 ± 10.8	57.16 ± 7.7	60.38 ± 13.9	56.8 ± 09.4
	More than high school	*62.68 ± 11.2	*60.84 ± 8.8	*65.08 ± 13.5	*59.0 ± 10.1

*statistically significant.

# ANOVA with post hoc comparisons

## Independent T test

Table 4 shows that current marital and earning status was significant predictor for HRQoL among the current smokers. The respondents who were currently married and earning had significantly higher QoL scores in physical domain while currently earning respondents had better QoL scores in psychological and social domains well. Socio–demographic variables did not predict QoL scores in the environmental domain.

**Table 4:** Predictors of HRQoL score among current smokers of the study. (n = 125).

Variables	Physical		Psychological		Social		Environmental	
	AOR	95% CI	AOR	95% CI	AOR	95%CI	AOR	95%CI
**Age**	- 0.043	-3.46–2.33	- 0.17	-3.87–0.53	0.018	-3.00–3.53	- 0.102	-3.62–1.42
**Sex**	- 0.100	-7.33–2.48	0.046	-2.91–4.56	-0.11	-8.54–2.53	- 0.013	-4.53–4.00
**Marital Status**	- 0.222	-19.6 - -0.56*	-0.023	- 8.04–6.47	-0.13	-17.87–3.61	-0.09	-11.8–4.73
**Earning status**	- 0.287	-11.09–2.48*	-0.240	-7.4 - -0.90*	-0.31	-13.33- -3.63*	- 0.157	-6.75–0.73
Education	0.057	-2.00–3.54	0.056	-1.55–2.66	0.04	-2.38–3.86	0.003	- 2.37–2.44

*statistically significant; multivariate linear regression

Among the non- smokers, education was the significant predictor of HRQoL among non–smokers. The results show that non—smokers with higher educational status had better QoL scores in all the four domains. Also, married non-smokers had better HRQoL in social domain. (Table 5)

**Table 5:** Predictors of HRQoL score among non-smokers in the current study. (n = 125).

Variables	Physical		Psychological		Social		Environmental	
	AOR	95% CI	AOR	95% CI	AOR	95%CI	AOR	95%CI
**Age**	-0.126	-3.53–0.97	0.029	-1.66–2.16	-0.07	-4.01–2.11	0.148	-0.81–4.05
**Sex**	-0.147	-8.23–0.99	-0.131	-6.65–1.17	0.03	-5.22–7.30	- 0.007	-5.1–4.79
**Marital Status**	-0.108	-9.50–3.00	-0.141	- 8.91–1.68	-0.24	-18.10 - -1.13*	-0.173	-12.4–1.07
**Occupation**	-0.088	-6.35–2.28	-0.085	-5.33–1.98	0.14	-1.35–10.36	0.070	-2.88–6.41
**Education**	0.222	0.19–5.19*	0.286	0.82–5.06*	0.25	0.73–7.51*	0.417	2.78–8.17*

*statistically significant

#multivariate linear regression.

## Discussion

This study was done to establish an association between cigarette smoking and HRQoL by using a sample from Nepalese population. We found that study patients smoked cigarettes for an average duration of 17 years while, the average quantity of cigarettes smoked were 4 sticks per day. This is in contrast to other studies which reported lower duration[24] and use of higher number of smoked tobacco products.[25]Among the smokers, 08.8% per cent of patients also used smokeless tobacco. We also found significantly higher percent of smokers consumed alcohol compared to never smokers, which was similar to studies done in Spain[26] and Brazil[24]. Furthermore, majority of the current smokers in the study belonged to higher age group, were males, currently married and earning.

In the current study, though never smokers had higher mean QOL scores, differences in all the 4 domains were relatively small and thus, cigarette smoking failed to be a significant predictor of HR-QOL. The existing evidence in this context seemed to vary in various epidemiological studies. Similar findings were reported by Japanese and Finnish population based surveys which observed insignificant differences in HR-QOL between daily smokers compared to non-smokers.[18,26] Some studies have reported no difference in HR-QOL among current smokers and ex-smokers as well.[27–29] One study also concluded that short-term differences in HRQOL for smokers and nonsmokers are not relevant.^4^ On the other hand, large number of studies conclude current smokers to have poorer QOL compared to ex-smokers and never smokers.[5,10,11,25] One of the explanations could be that majority of the sampled current smokers turned out to be light smokers. The other explanation could be perception of smoking among the Nepalese population. A study showed that Nepalese young adults expressed various benefits of smoking which included enjoyment, stress reliever, relaxation technique and comfortable around peers.[30] Another study among the Nepalese adolescents also showed that there are social benefits attached to smoking.[31]

We found that various socio-demographic characteristics of patients were significant predictors of HRQoL. In the current study, males had significantly higher HRQol scores compared to women in the physical domain. Prior study also proved that association of smoking with impaired quality of life is more marked in females than in males.[32] Also, patients who were currently earning had higher scores in physical, psychological and social domains while, patients with more than high school education had better HRQoL in all the four domains. This is similar to a Finnish study, which reported significant association of gender, income, education, marital status with HRQoL.[18] Another study concluded that other socio-economic characteristics are better indicators of quality of life than smoking status.[29,30,31,33]

Currently earning smokers had higher HRQoL scores in physical, psychological and social domains. On the other hand, higher education was a predictor of better HRQoL among the non-smokers. However, factors like sex, ethnicity and education were found to be associated with HRQoL among smokers in other epidemiological studies. For example, a study from Belgium showed lower HRQoL scores among female smokers with low education level.[34] Along with this, smokers from Hispanic ethnicity have also shown lower HRQoL compared to the non-Hispanic white counterparts.[35]

We tried to ensure comparability between the two groups by selecting patients who came for similar dental treatment to the hospital. However, we recognize various limitations of the study. Firstly, since we used non-probability sampling, leading to selection bias and limited external validity. Socially desirable responses on duration and amount of smoking might have led to reporting bias.

## Conclusion

According to study results, relationship between smoking status and self reported QoL is unclear. In this Nepalese sample, socioeconomic variables like gender, earning status and education were determinants of HRQoL rather than smoking status. Thus, the policy makers should also focus on wider determinants of ill health and well being and not just current use of smoking. Further research, using an improved study design, is still needed to understand the effect of smoking on perceived HRQoL.

## Supporting information

S1 Raw Dataset (XLSX)
